# Assessing the Efficacy of Antibiotic Therapy: A Retrospective Study Comparing 875 mg vs. 500 mg of Amoxicillin/Clavulanic Acid for the Management of Acute Apical Abscesses

**DOI:** 10.3390/dj14020071

**Published:** 2026-01-26

**Authors:** Tal Capucha, Shaul Lin, Dani Noy, Chaim Ohayon, Mordechai Grupper, Daniel Moreinos, Marc Rothman, Dekel Shilo, Omri Emodi, Adi Rachmiel, Roni Dakar

**Affiliations:** 1 Department of Oral and Maxillofacial Surgery, Rambam Health Care Campus, Haifa 3109601, Israel; capuchatal@gmail.com (T.C.); dr.dani.noy@gmail.com (D.N.); haim.ohayon@mail.huji.ac.il (C.O.); dekelshi@yahoo.com (D.S.); omri.emodi@gmail.com (O.E.); rach_adi@netvision.net.il (A.R.); 2 The Ruth and Bruce Rappaport Faculty of Medicine, Technion—Israel Institute of Technology, Haifa 3525433, Israel; ronidakar@gmail.com; 3 Department of Endodontics and Dental Trauma, Rambam Health Care Campus, Haifa 3109601, Israel; 4 Infectious Disease Unit, Rambam Health Care Campus, Haifa 3109601, Israel; moti.grupper@gmail.com; 5 Department of Endodontics, Galilee College of Dental Sciences, Galilee Medical Center, Nahariya 22100, Israel; dmoreinos@gmail.com; 6 Oral and Maxillofacial Surgery Division, Albert Einstein Medical Center Philadelphia, Philadelphia, PA 19107, USA; rmarcg@comcast.net

**Keywords:** amoxicillin-potassium clavulanate combination, blood cell count, c-reactive protein, drug administration schedule, periapical abscess

## Abstract

**Introduction**: Antibiotics are routinely prescribed for odontogenic abscesses in emergency departments and dental offices. Augmentin is recommended for moderate to severe dentofacial infections. It is usually prescribed in two popular regimens, namely twice (bid) or three times (tid) per day. The aim of this study was to compare the efficacy of two different formulations of amoxicillin–clavulanate, 875/125 mg bid versus 500/125 mg tid, for the treatment of acute dental apical abscesses with orofacial involvement. **Methods**: Sixty-one patients with acute apical abscesses were prescribed Augmentin in either an 875/125 mg bid or 500/125 mg tid formulation. The patients were tested for inflammatory markers upon admission and again after 72 h. **Results**: Although all patients experienced a decrease in inflammatory markers over 72 h of antibiotic therapy, there was a statistically significant greater decrease in white blood cells and neutrophils in the patients receiving the 500/125 mg tid regimen. **Conclusions**: A 500/125 mg tid Augmentin regimen results in a greater decline in inflammatory markers than 875/125 mg bid over 72 h in the setting of dentofacial infection.

## 1. Introduction

The use of antibiotics is widespread among the general population, particularly in cases of severe dental orofacial infections [1,2]. Dentists account for approximately 7% of antibiotic prescriptions, and dentists have been reported to prescribe antibiotics with inappropriate frequency and for longer periods than recommended [3]. Inappropriate prescribing has also been reported for conditions such as pulpitis, dry socket, and chronic periodontal lesions, which require operative intervention rather than antibiotics [4]. A British study examining the appropriate use of antibiotics in emergency dental care found that the majority of patients presenting with pain had either pulpitis or localized dental abscesses. Retrospective analysis revealed that 75% of the patients were inappropriately prescribed antibiotics. Amoxicillin was given in 72% of cases [5]. Some emergency centers routinely administer IV antibiotics. However, systemic antibiotics should be used with restraint due to the possibility of allergic reactions, toxicity, side effects, and the development of microbial resistance [6,7].

In a study evaluating the healing time of acute apical abscesses, where 618 patients were diagnosed with acute apical abscesses and prescribed different types of antibiotics, aminopenicillins (mainly amoxicillin) and amoxicillin–clavulanate antibiotics were found to reduce the healing time to less than 6 days, as compared to other antibiotics, for which the healing time exceeded 6 days [8]. When amoxicillin is given to patients with acute apical abscesses, improvements in signs and symptoms are reported as soon as three days of treatment [9]. Aminopenicillins and amoxicillin–clavulanate remain the antibiotics of choice in patients with no history of allergic reactions to penicillin [1,8,10,11]. As an alternative, the addition of a β-lactam and β-lactamase inhibitor (e.g., amoxicillin–clavulanate) may be considered due to the increasing resistance to aminopenicillins alone [11,12].

Clavulanic acid enhances the antibacterial spectrum of amoxicillin by rendering most β-lactamase-producing isolates susceptible to the drug. In clinical trials amoxicillin–clavulanic acid was shown to be clinically and bacteriologically superior to amoxicillin alone and at least as effective as numerous other comparable agents, such as orally administered cephalosporins, cotrimoxazole, doxycycline, and bacampicillin, in the treatment of adults and children with the most common forms of odontogenic infections [10,13]. Clavulanic acid alone possesses only weak antibacterial activity [13]. However, the addition of clavulanic acid to amoxicillin enhances the susceptibility of amoxicillin-resistant strains, which include *Staphylococcus aureus* (but not methicillin-resistant strains), *Haemophilus* spp., *Branhamella catarrhalis*, *Neisseria gonorrhoeae*, *Escherichia coli*, *Proteus* spp., *Klebsiella pneumoniae*, *Citrobacter diversus*, *Salmonella* and *Shigella* spp., *Campylobacter jejuni*, *Bacteroides* spp., and *Mycobacterium* spp. [13].

The European Society of Endodontology recommends that the way to successfully manage acute apical abscesses is to adequately debride the infected root canal and thereby remove the pathogenic microorganisms and their byproducts [10,14,15]. When orofacial spaces are involved, incision and drainage should be performed [1,2]. Antibiotic treatment is indicated after flare-ups, after the diffusion of orofacial cellulitis, or when debridement cannot be performed due to systemic involvement such as trismus [16,17]. Systemic (IV administration) antibiotics may be indicated in such severe cases in order to reduce further spreading of the infection [10,11,18,19,20,21].

A common means to evaluate patient health in severe odontogenic infection is taking a “complete blood count” (CBC), also known as a “full blood count” (FBC), that can detect diseases, inflammation, autoimmune disorders, anemia, bone marrow disorders, infections, etc. [22]. A CBC is a blood test designed to evaluate a patient’s health status. Not only does it include quantitative evaluations of white blood cells (WBCs), erythrocytes, platelets, and more, but it can also provide qualitative information on the chemistry and biochemistry that are constituents of the blood. The cells that are analyzed are red blood cells (erythrocytes) and WBCs including neutrophils, eosinophils, basophils (leucocytes), lymphocytes, monocytes, and platelets. CBCs are an effective tool to evaluate a patient’s reaction to antibiotic treatment. This is accomplished by repeated CBCs and assessment of the improvement in the WBC counts [22,23,24,25] (Table 1).

C-reactive protein (CRP) is an acute-phase-reactant plasma protein that elevates markedly in response to inflammation or infection. CRP is synthesized in hepatocytes and is regulated by IL-6 [26,27]. During an inflammatory state there is a rapid increase in the synthesis of CRP, within hours, as part of the innate immune response [28]. A healthy adult with no evidence of inflammation has a CRP level of less than 0.3 mg/L. The CRP levels fall and rise with the rapid onset and removal of the inflammatory stimulus, respectively. Those with chronic infections or chronic inflammatory diseases, like rheumatoid arthritis, can exhibit persistently elevated CRP levels. Elevated CRP can have various causes such as acute and chronic conditions or infections, and diseases of non-infectious origin [29]. In most cases, high CRP levels signify an infectious etiology [30]. Females and elderly patients tend to exhibit higher CRP levels. Mild elevations in CRP, potentially influenced by obesity, insomnia, depression, smoking, and diabetes, can also occur independently of systemic or inflammatory diseases. Certain medications, like statins or non-steroidal anti-inflammatory drugs (NSAIDs), can also affect CRP levels. It is well known that statins can lower CRP values. Therefore, interpretations of results in individuals with these comorbidities or on these medications should be carefully considered [31,32,33].

Normal neutrophil counts vary within 1450–7500 per microliter. Neutrophilia is defined as more than 7500 neutrophils per microliter. When infection is suspected as the primary cause of the inflammation, there is an immunological response of leucocyte production, in which case neutrophils may represent 50–70% of all circulating leucocytes. Neutrophils contain 3–5 nuclei and numerous granules containing an enzyme named lysosome. The lysosome breaks down the bacteria into small components after the neutrophil engulfs the bacteria. This is known as phagocytosis [16]. The neutrophil can also attach to bacteria through the FC fragment of antibodies to engulf the bacteria [34]. The neutrophil count can also be influenced by systemic diseases such as myelofibrosis and leukemia. Antipsychotic medications, such as clozapine, can also have an effect on the total count [35,36].

An acute apical abscess is usually confined to intraoral spaces, known as localized infection, but occasionally may spread to deeper fascial planes in the head and neck, resulting in severe complications and mortality [1,2,9,37,38]. In severe cases, treatment involves surgical drainage and systemic (IV) antibiotics [2,9,10,21]. However, the empirical treatment by means of oral antibiotics, most commonly used in cases of acute apical abscesses, is amoxicillin–clavulanate. It may be prescribed in two commercially available formulations, 875 mg tablets (Augmentin^®^, GlaxoSmithKline, London, UK) prescribed twice a day (bid: bis in die) and amoxicillin–clavulanate 500 mg tablets (Augmentin^®^, GlaxoSmithKline, London, UK) prescribed three times a day (tid: ter in die) for up to 7 days. The aim of this study is to compare the efficacy of the two available formulations of amoxicillin–clavulanate, namely 875 mg twice a day versus 500 mg three times a day, for the treatment of acute apical abscesses.

## 2. Materials and Methods

The studied sample consisted of a retrospective cohort of patients who attended the emergency department and were diagnosed with an acute apical abscess between January 2016 and November 2023. This study was approved by the medical ethics committee (approval number RMB-D-0537-23). All data were extracted retrospectively from the electronic medical record and fully anonymized prior to analysis. No changes were made to patient management as part of this study, and no patients were contacted specifically for research purposes. The inclusion criteria were healthy patients with acute apical abscesses and orofacial swelling that included infection in the primary spaces with no fever. An additional inclusion criterion required no allergy to amoxicillin. All included patients were treated with oral antibiotics, received intraoral incision and drainage, and returned for follow-up. The exclusion criteria included patients who had taken antibiotics within 14 days prior to attending the emergency department and patients with chronic diseases, with a history of malignancy, or who had received a bone marrow transplant. Furthermore, patients who required treatment via extraoral incision and drainage, those who developed an allergic reaction or severe abdominal pain during antibiotic treatment, and those who received intravenous antibiotics, corticosteroids, NSAIDs, statin therapy, or immunosuppressant drugs that alter CBC counts were excluded as well. Throughout the study period (2016–2023), the diagnostic criteria for acute apical abscess, the standard drainage protocol, and the institutional recommendations for oral amoxicillin–clavulanate dosing remained unchanged in the department protocol. All blood tests were processed in the same hospital laboratory, using standardized automated analyzers, and reference ranges did not change during this time.

The treatment on day one in the emergency department comprised patient intake which included documenting the patient’s medical history, age, sex and a systemic health evaluation that consisted of blood pressure, body temperature, CRP, and a CBC. If acute apical abscesses emerged from the medical anamnesis, a panoramic radiograph was ordered for further evaluation. A radiograph taken together with CBC results can identify whether an infection is the etiology of systemic health deterioration.

### 2.1. Drainage

After the clinical and radiographic evaluation, an intraoral subperiosteal drainage was performed on day one (T0), under local anesthetic of Lidocaine HCL 2% and Epinephrine 1:100,000 (Novocol, Cambridge, ON, Canada). A number 10 (Swan-Morton, Sheffield, UK) blade was inserted at the tip of the inflamed tissue until it reached the bone. The wound was copiously irrigated with 20 mL of sterile saline solution (Braun AG, Melsungen, Germany). A Penrose drain (a flexible tube that drains fluid from a surgery site; Changzhou Yuekang Medical, Changzhou, China) was inserted and fixed for 3 days with a 3/0 silk suture (Dochem, Shanghai, China). Patients were instructed to begin the antibiotic regimen immediately after discharge from the department and to take the tablets at evenly spaced intervals according to the prescription. Adherence to the prescribed regimen was assessed at the 72 h follow-up visit. Patients were asked whether they had started the medication as instructed, whether they had missed any doses, and whether they had used any additional medications. After three days, under local anesthetic of Lidocaine (HCL 2% and Epinephrine 1:100,000), the sutures and the drain were removed from the mouth.

### 2.2. Study Groups

The antibiotics administered were “open labels”, indicating that patients knew the type of antibiotics they were taking.

(1) Group 1 (G1): Patients were prescribed amoxicillin–clavulanate (Augmentin^®^, GlaxoSmithKline, London, UK): 875/125 mg twice daily for 7 days (see File S1).
(2) Group 2 (G2): Patients were prescribed amoxicillin–clavulanate (Augmentin^®^, GlaxoSmithKline, London, UK): 500/125 mg three times daily for 7 days (see File S2).

After three days the patients were instructed to return for a follow-up. The patients underwent additional assessments, including repeated CRP and CBC tests and drain removal. A clinical and radiographic summary was given to the patients with recommendations for ongoing dental care, which could involve either root canal treatment or tooth extraction.

### 2.3. Statistical Analysis

Descriptive statistics in terms of the mean ± SD were calculated for all parameters in this study. Differences between the two groups (amoxillin–clavulanate 875 mg vs. amoxillin–clavulanate 500 mg) for continuous parameters (age, WBCs, CRP, neutrophils) were assessed with the *t*-test or Mann–Whitney U test, as appropriate. The Chi-square or Fisher exact test was used for categorical parameters (e.g., gender).

Multivariable general linear models were implemented to explore the difference between the study groups after adjusting for the baseline value, age, and gender. *p* < 0.05 was considered significant. SAS 9.4 software (SAS Institute Inc., Cary, NC, USA) was used for all statistical analysis.

## 3. Results

During the study period, a total of 2377 patients presented to the emergency department with a diagnosis of acute apical abscess. Of these, 2316 patients did not meet the inclusion criteria or were excluded due to one or more predefined exclusion criteria. The most frequent reasons for exclusion were recent antibiotic use, the presence of significant systemic comorbidities, or the need for intravenous antibiotics and/or extraoral incision and drainage.

### 3.1. Experimental Groups

Clinical and demographic data were obtained from the database registry. All patients were monitored for their complete blood count (CBC) and C-reactive protein (CRP) levels upon admission (T0) and after 72 h (T1). Sixty-one patients met all inclusion criteria and were included in the present study: Group one (G1) included 35 patients (12 (34%) males and 23 females (66%)) with a mean age of 48.1 ± 12.7 years, who were prescribed amoxicillin–clavulanate 875 mg bid for 7 days. Group two (G2) comprised 26 patients (9 males (35%) and 17 females (65%)) with a mean age of 46.5 ± 17.4 years, who were prescribed 500 mg amoxicillin-clavulanate tid for 7 days. Summary demographic and baseline laboratory values for WBCs, neutrophils, and CRP at baseline (T0) are presented in Table 2. No significant differences were found among the study groups regarding demographic or baseline laboratory results.

### 3.2. White Blood Cells

**Figure 1:**
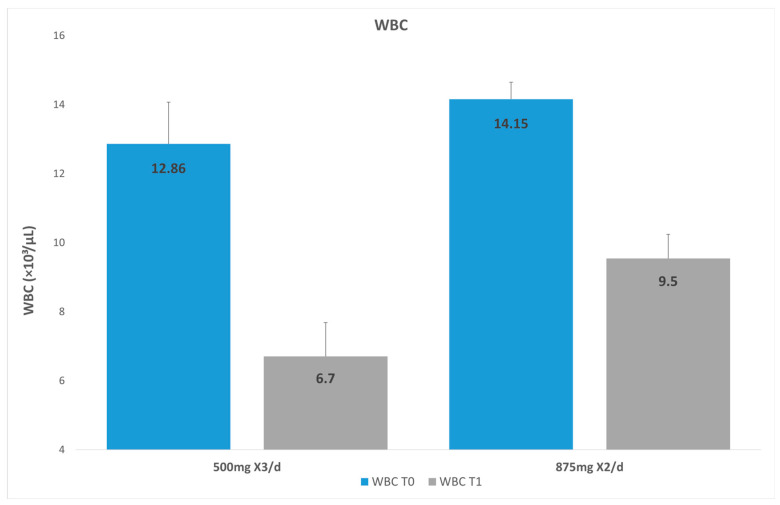
White blood cells in group 1 versus group 2.

The average results for the WBC count were as follows: For G1 on day one (T0), the mean WBC count was 14.16 × 10^3^/µL, and after 3 days (T1) it was 9.54 × 10^3^/µL. For G2 on day one (T0), the WBC count was 12.86 × 10^3^/µL, and after 3 days it was 67 × 10^3^/µL (Figure 1). Both groups showed a significant decrease in the WBC count between T0 and T1 (*p* < 0.001). For G2 the mean WBC after 72 h was significantly lower compared to that for G1 (*p* = 0.0012; Table 3). After adjusting for age, gender, and baseline WBCs, the difference between the groups remained statistically significant (*p* = 0.0042; adjusted mean for G1: 9.26 [95%CI: 8.11–10.4] and adjusted mean for G2: 6.71 [95%CI: 5.4–8.0]).

### 3.3. Neutrophils

**Figure 2:**
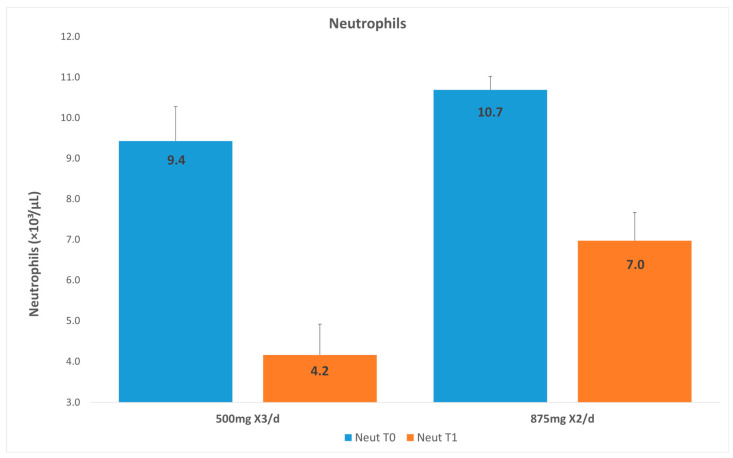
Neutrophils in group 1 versus group 2.

The neutrophil count at T0 in G1 was 10.7 × 10^3^/µL, and that at T1 was 7.0 × 10^3^/µL. The neutrophil count at T0 in G2 was 9.43 × 10^3^/µL, and that at T1 was 4.2 × 10^3^/µL (Figure 2). Both groups showed a significant decrease in the neutrophil count between T0 and T1 (*p* < 0.0001). For G2 the mean neutrophil count after 72 h was significantly lower compared to that for G1 (*p* = 0.0006; Table 3). After adjusting for age, gender, and baseline neutrophil count, the difference between the groups remained statistically significant (*p* = 0.0014; adjusted mean for G1: 6.61 [95%CI: 5.74–7.48] and adjusted mean for G2: 4.43 [95%CI: 3.44–5.42]).

### 3.4. C-Reactive Protein

**Figure 3:**
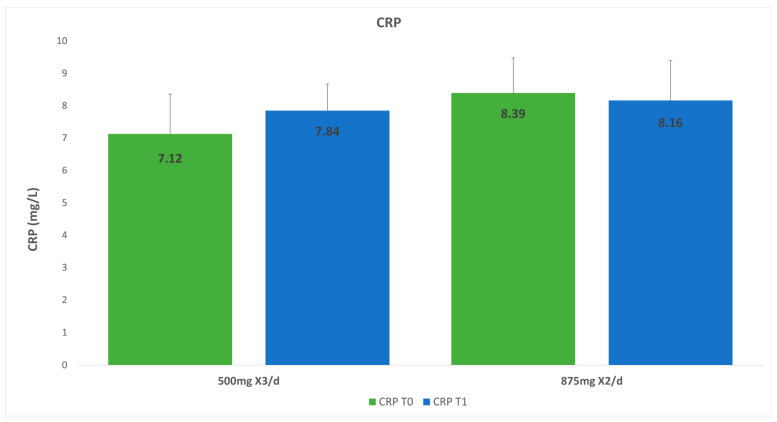
C-reactive protein in group 1 versus group 2.

The results for G1 showed CRP counts of 8.41 mg/L at T0 and 8.27 × 10^3^/µ mg/L at T1. The CRP count in G2 on day one (T0) was 7.1 mg/L, and that after 3 days was 7.8 mg/L. The change in CRP from T0 to T1 showed no significant difference for either group (mean difference for G1 was 0.13 with SD = 7.83, *p* = 0.9393 and mean difference for G2 was −0.71 with SD = 2.02, *p* = 0.0923).). Also, no significant difference was found in the CRP count at 72 h between the groups (*p* = 0.8572; Figure 3, Table 3).

## 4. Discussion

The null hypothesis of this study was that no difference exists between two commonly prescribed oral amoxicillin–clavulanate regimens (875/125 mg Tid and 500/125 mg Bid) in terms of their effect on systemic inflammatory markers in patients with acute apical abscesses. This hypothesis was partially rejected. While both regimens were effective and resulted in significant reductions in WBC and neutrophil counts after 72 h, the 500/125 mg regimen was associated with significantly lower averages of these markers at 72 h. No significant differences were observed in CRP levels, which can be attributed to limitations of this study. Although all patients experienced a decrease in inflammatory markers over 72 h of antibiotic therapy, there was a statistically significantly greater decrease in white blood cells and neutrophils in the patients receiving the 500/125 mg tid regimen, resulting in a greater decline in inflammatory markers than 875/125 mg bid over 72 h in the setting of dentofacial infection.

Antibiotics are important drugs that can save lives in the case of severe infections caused by acute apical abscesses [39]. These infections can spread (cellulitis) into deep neck spaces and lead to potentially lethal complications such as Ludwig’s angina [40]. Since the use of antibiotics became common in the treatment of odontogenic infections, the death toll from such infections has been reduced from 50% to 8% [41,42]. Because of the perceived safety net with antibiotic treatment, dentists are over-prescribing. Over time, increasing evidence indicates that over-prescription provokes the long-term persistence of bacteria that are resistant to antibiotics [39,43,44]. As resistance to aminopenicillins rose, newer antibiotics designed to overcome this resistance were developed. Amoxicillin–clavulanate (Augmentin^®^) has been found to reduce resistance of bacteria to amoxicillin, a drug that has traditionally served as the drug of choice in odontogenic infections [8,10,11,21,31].

A complete blood count is used to assess WBC counts following antibiotic treatment. This blood test can provide the medic or dentist with data about a patient’s health [23,24,25]. When an infection is present, WBCs that are in the bone marrow are secreted into the blood as a consequence of stimulation from the zone of inflammation. WBCs, CRP, and neutrophils are part of the innate immune system and are present in large numbers in the blood when inflammation and infections such as acute apical abscesses are present [10]. The most common factors indicative of infection are neutrophils. Neutrophils are responsible for fighting off bacterial infection. Elevated CRP is not specific to bacterial infections and can also be present in non-infectious diseases, making it a less reliable marker. Furthermore, medications such as statins and NSAIDs, especially Naproxen, are linked to a marked reduction in CRP levels, additionally making them less reliable [45].

In this study two different formulations of amoxicillin–clavulanate were compared, i.e., 875 mg bid mg versus 500 mg tid, via CBCs assessed at T0 and T1. This time frame correlates with the clinical improvement in signs and symptoms after 72 h in patients with acute apical abscesses [1,9]. The results showed that the concentrations of WBCs and neutrophils specifically were similar in both groups at T0, and after three days the two groups showed significant decreases in the concentrations of WBCs and neutrophils; this indicates that amoxicillin–clavulanate is an effective antibiotic at both doses of 875 mg and 500 mg. Nonetheless, in G2 (500 mg tid) the decline in neutrophils was significantly greater than that in G1 (*p* = 0.003). The number of neutrophils in the blood is directly correlated to the severity of bacterial infection, since the immune system is activated by bacteria and byproducts that promote Toll-like receptors to secrete cytokines that stimulate the formation and migration of neutrophils into the blood and then into the infected tissue. The 500 mg tid formulation of amoxicillin–clavulanate successfully helped the immune system fight bacterial infection better than the 875 mg formulation of amoxicillin–clavulanate. This result can be attributed to the pharmacodynamics of the antibiotic. Amoxicillin is a beta-lactam; thus, its efficacy against bacteria is by means of a time-dependent mechanism, namely, the time for which the free concentration of the antibiotic remains above the minimal inhibitory concentration (MIC) of the bacteria within each dosing interval [46,47,48,49,50]. It was established that this parameter should be at least 40–50% of the dosing interval for maximal efficacy of a beta-lactam [43]. When amoxicillin–clavulanate is administered every 8 h, 50% of the dosing interval is 4 h, as opposed to when it is administered every 12 h, when 50% of the interval is, of course, longer at 6 h. Increasing the dose of a beta-lactam has less of an effect on the time above the MIC than lengthening the dosing interval. Consequently, it is highly plausible that the free concentration of amoxicillin in the serum and tissues will stay above the MIC for a longer portion of the dosing interval when the dosing interval is shorter, thus being more efficacious when administered every 8 h as opposed to every 12 h [43,47,48,49,50].

C-reactive protein (CRP) is a nonspecific inflammatory marker that may be elevated in response to infection but also in cases of trauma or surgical intervention. Therefore, CRP is not specific to bacterial infections [29,33,46]. In the present study, no significant differences in CRP levels were observed between the two groups or between time points T0 and T1. However, a slight increase in CRP was noted in Group 1 at T1, which may be attributed to the surgical trauma associated with the incision and drainage procedure and to variability among different clinicians performing the surgical procedure. Since it is impossible to quantify the effect of the surgical procedure on CRP, the results show a limitation. Furthermore, some CRP measurements were N/A for some participants, which may limit the generalizability of the findings related to this parameter and result in different observations in future studies.

In acute apical abscesses the infection can spread to different spaces in the vicinity of the apexes of the upper and lower teeth. The abscesses usually spread to orofacial spaces according to their relationship to endodontic infections (Table 4). There are two types of facial spaces: “*Primary spaces*” and “*Secondary spaces*” (also known as indirect). The primary spaces include the “canine space” and “vestibular space”. The secondary spaces include the “pterygomandibular space” and “Para-pharyngeal (Lateral and Retro) spaces”. Infection spreads from the primary to the secondary spaces, which are deeper in the head and neck spaces [51,52]. This study therefore only included systemic infection of primary spaces since involvement of secondary spaces required patients to receive intravenous antibiotics and often be hospitalized for surveillance.

Huttner et al. (2020) appear to agree with our findings in their conclusions where they state that “to achieve sufficient amoxicillin and high clavulanic acid exposure, the optimal regimen is to administer narrower ratio amoxicillin-clavulanic acid in a three times daily regimen” [13]. Contrary to our findings, however, a comparison between 875 mg bid and 500 mg tid of amoxicillin–clavulanate in the treatment of acute sinusitis did not reveal any statistical differences between the two doses in the improvement of symptoms [53]. This finding could be attributed to the difference in the quantities of bacteria involved in these diseases. In acute bacterial maxillary sinusitis, 50% of bacterial infections consist of *Streptococcus pneumonia* and *Hemophilus* [53,54]. On the other hand, acute apical abscesses are caused by a large number of Gram-negative and Gram-positive bacterial species. In fact, in aspirates of acute apical abscesses in forty-two patients, as many as 81 species, particularly Gram-negative and some Gram-positive strains, were found [1,14,21].

### Limitation

This study has some limitations that should be acknowledged. First, this was a retrospective study that compared two interventions. Due to a lack of randomization and blinding, the study groups may not have been balanced; therefore, confounding factors (such as demographic and baseline values, as well as infection severity, host immune response, or medication adherence) may have biased the results. However, as mentioned, this was a retrospective study; therefore, unlike in randomized controlled trials, the allocation of the treatments represents the actual allocation that occurs in real life. Thus, its generalizability is higher. Overall, the study groups were balanced regarding age, gender, and baseline laboratory values. In order to control for these factors, multivariable models were implemented to adjust for baseline values and demographic variables (age and gender). Second, although no formal sample size calculation was made prior to the study data collection, post hoc power calculations (based on the obtained data) demonstrated power > 90% for both WBCs and neutrophils. CRP had very low statistical power (<10%) in addition to having imbalanced missing observations among the study groups. Finally, serum antibiotic levels were not measured, as this study was not designed as a pharmacological investigation. Consequently, the interpretation of the findings is based on clinical and laboratory outcomes rather than direct pharmacokinetic assessment.

## 5. Conclusions

Severe dental infections such as acute apical abscesses are polymicrobial infections. Reducing the dose interval for par/oral amoxicillin–clavulanate improves the antibacterial effects of the antibiotics. A formulation of 500 mg tid shows superior outcomes to 875 mg bid in the treatment of acute apical abscess.

## Figures and Tables

**Table 1:** Normal values for white blood cell counts.

Leucocytes	Mean Number Per mm^3^	Normal Range
White blood cells	7400	4500–11,000/mm^3^
Neutrophils	4400	40–60%
Eosinophils	300	1–4%
Basophils	40	<1%
Lymphocyte	2500	20–40%
Monocytes	300	2–8%

**Table 2:** Demographic information and baseline laboratory values.

	875/125 mg Bid (G1)	500/125 mg Tid (G2)	*p*-Value
Age	48.1 ± 12.7 (n = 35)	46.5 ± 17.4 (n = 26)	0.6880
Gender—male	12 (34.3%)	9 (34.6%)	0.9786
Baseline laboratory			
WBCs	14.2 ± 6.2 (n = 35)	12.9 ± 6.3 (n = 26)	0.4266
Neutrophils	10.7 ± 4.9 (n = 35)	9.4 ± 4.4 (n = 26)	0.3076
CRP	8.4 ± 5.3 (n = 25)	7.1 ± 6.4 (n = 25)	0.2365

Mean ± standard deviation.

**Table 3:** Study endpoints.

Parameter	875/125 mg Bid (G1)	500/125 mg Tid (G2)	*p*-Value
WBC (×10^3^/µL)	9.54 ± 3.96 (n = 35)	6.70 ± 2.56 (n = 26)	0.0012
Neutrophils (×10^3^/µL)	6.97 ± 4.02 (n = 35)	4.16 ± 1.73 (n = 26)	0.0006
CRP (mg/L)	8.16 ± 7.34 (n = 27)	7.85 ± 5.65 (n = 25)	0.8572

Mean ± standard deviation.

**Table 4:** Facial space involvement in endodontic infection.

Mode of Involvement
**Primary maxillary spaces** **Secondary spaces (Indirect)**
Canine space Masseteric space
Buccal space Pterygomandibular space
Infratemporal space Temporal (Superficial and Deep) spaces
Buccal space Pre-vertebral space
Sub-mental space Para-pharyngeal (Lateral and Retro) spaces
Sub-mandibular space
Sub-lingual space

## Data Availability

The data supporting the findings of this study are not publicly available due to ethical restrictions.

## Supplementary Materials

The following supporting information can be downloaded at https://www.mdpi.com/article/10.3390/dj14020071/s1, File S1: Amoxicillin/Clavulanic acid 875 mg twice per day. File S2, Amoxicillin/Clavulanic acid 500 mg three times per day.
