# Supplementary material for: Assessing the Efficacy of Antibiotic Therapy: A Retrospective Study Comparing 875 mg vs. 500 mg of Amoxicillin/Clavulanic Acid for the Management of Acute Apical Abscesses
Source: Dent J (Basel). 2026 Jan 26;14(2):71. doi: 10.3390/dj14020071 (PMC12938990; doi:10.3390/dj14020071)
Supplement: Supplementary file 1 [file dentistry-14-00071-s001.zip › Group 2.docx]

| **Patient (N)** | **Tooth** | **Age** | **SEX** | **WBC (T0)** | **Neutrophils (T0)** | **CRP (T0)** | **WBC (T1)** | **Neutrophils (T1)** | **CRP (T1)** |
| --- | --- | --- | --- | --- | --- | --- | --- | --- | --- |
| *1* | 37 | 28 | M | 13.7 | 7.6 | 0.8 | 7.9 | 3.6 | 0.9 |
| *2* | 37 | 30 | F | 14.1 | 11.2 | 6.2 | 8.2 | 5.3 | 7.4 |
| *3* | 35 | 42 | F | 11.9 | 8.7 | 2.7 | 5.8 | 3.0 | 2.0 |
| *4* | 41-42 | 71 | F | 19.1 | 14.8 | 14.0 | 11.1 | 7.1 | 12.0 |
| *5* | 36 | 22 | M | 11.5 | 8.8 | 4.4 | 5.8 | 3.4 | 4.6 |
| *6* | 25 | 49 | F | 11.5 | 9.4 | 3.9 | 5.9 | 3.4 | 4.0 |
| *7* | 14 | 30 | F | 9.4 | 7.4 | 4.2 | 4.5 | 3.6 | 5.0 |
| *8* | 35 | 56 | M | 11.0 | 8.4 | 4.0 | 8.6 | 6.2 | 4.4 |
| *9* | 23 | 55 | F | 19.2 | 13.0 | 2.0 | 9.3 | 5.0 | 3.2 |
| *10* | 13 | 43 | F | 12.5 | 8.5 | 2.7 | 6.0 | 3.4 | 3.2 |
| *11* | 45 | 29 | M | 14.4 | 11.5 | 1.1 | 6.9 | 4.6 | 1.3 |
| *12* | 25 | 59 | F | 10.8 | 7.7 | 7.8 | 5.2 | 3.1 | 9.4 |
| *13* | 23 | 78 | F | 8.8 | 6.7 | 10.7 | 4.2 | 2.7 | 15.0 |
| *14* | 25 | 46 | M | 17.2 | 14.3 | 5.5 | 8.3 | 5.7 | 7.7 |
| *15* | 35 | 58 | F | 9.8 | 7.7 | 5.0 | 4.7 | 3.1 | 7.0 |
| *16* | 23 | 85 | M | 15.5 | 12.9 | 16.0 | 7.4 | 4.9 | 15.0 |
| *17* | 37 | 61 | F | 8.8 | 6.2 | 3.6 | 3.5 | 2.3 | 5.0 |
| *18* | 38 | 48 | F | 12.9 | 9.0 | 17.0 | 5.2 | 3.4 | 16.0 |
| *19* | 43 | 40 | M | 11.5 | 8.2 | 8.1 | 4.6 | 3.1 | 11.3 |
| *20* | 38 | 26 | F | 38.5 | 26.0 | 22.9 | 15.4 | 9.9 | 18.0 |
| *21* | 13 | 47 | F | 8.7 | 6.3 | 3.3 | 5.2 | 3.7 | 4.8 |
| *22* | 44 | 64 | M | 6.5 | 5.0 | 22.0 | 3.9 | 2.9 | 21.0 |
| *23* | 23 | 25 | F | 12.0 | 6.9 | 0.5 | 7.2 | 4.0 | 0.7 |
| *24* | 36 | 20 | M | 13.2 | 10.0 | 4.7 | 7.9 | 5.8 | 6.8 |
| *25* | 45 | 44 | F | 6.0 | 6.0 | 5.2 | 5.0 | 3.3 | 10.3 |
| *26* | 11 | 53 | F | 6.0 | 3.0 | n/a | 6.6 | 1.7 | n/a |

WBC- white blood cells; CRP- C-reactive protein; T0- day 1; T1- day 3; n/a- not applicable
