# Supplementary material for: Assessing the Efficacy of Antibiotic Therapy: A Retrospective Study Comparing 875 mg vs. 500 mg of Amoxicillin/Clavulanic Acid for the Management of Acute Apical Abscesses
Source: Dent J (Basel). 2026 Jan 26;14(2):71. doi: 10.3390/dj14020071 (PMC12938990; doi:10.3390/dj14020071)
Supplement: Supplementary file 1 [file dentistry-14-00071-s001.zip › Group 1.docx]

Group 1, Amoxicillin/Clavulanic acid 875 mg twice per day

| **Patient (N)** | **Tooth** | **Age** | **SEX** | **WBC (T0)** | **Neutrophils (T0)** | **CRP (T0)** | **WBC (T1)** | **Neutrophils (T1)** | **CRP (T1)** |
| --- | --- | --- | --- | --- | --- | --- | --- | --- | --- |
| *1* | 36 | 70.5 | F | 6.6 | 3.7 | 4.6 | 8.2 | 5.4 | n/a |
| *2* | 36 | 55.5 | F | 7.1 | 5.9 | 10.0 | 4.8 | 4.0 | 1.3 |
| *3* | 23 | 42 | F | 7.4 | 4.0 | 4.9 | 9.6 | 2.7 | 1.0 |
| *4* | 47 | 39 | F | 7.5 | 6.6 | 0.0 | 8.5 | 4.5 | 0.0 |
| *5* | 48 | 39 | F | 7.8 | 3.9 | n/a | 6.5 | 2.7 | n/a |
| *6* | 36 | 36 | F | 9.1 | 6.5 | 0.6 | 5.7 | 4.4 | 2.2 |
| *7* | 44 | 31.5 | F | 9.2 | 6.0 | 9.7 | 13.4 | 11.1 | 1.4 |
| *8* | 24 | 51 | F | 9.7 | 7.4 | 1.1 | 7.2 | 4.1 | 10.2 |
| *9* | 27 | 30 | M | 10.4 | 7.8 | n/a | 6.9 | 4.5 | 15.7 |
| *10* | 36 | 31.5 | F | 10.7 | 6.4 | 4.8 | 3.4 | 2.2 | 5.2 |
| *11* | 36 | 36 | F | 11.0 | 7.6 | 4.5 | 17.3 | 9.3 | 5.4 |
| *12* | 24 | 51 | F | 11.2 | 8.5 | 5.2 | 21.6 | 4.9 | 4.7 |
| *13* | 13 | 52.5 | M | 11.5 | 8.0 | 11.0 | 7.9 | 6.2 | n/a |
| *14* | 25 | 45 | F | 11.5 | 9.3 | n/a | 7.6 | 4.8 | n/a |
| *15* | 44 | 45 | M | 11.7 | 8.4 | 11.4 | 8.0 | 4.9 | 10.3 |
| *16* | 23 | 57 | M | 12.4 | 8.0 | 10.8 | 6.7 | 4.2 | 10.7 |
| *17* | 44 | 36 | F | 12.9 | 10.6 | 13.9 | 5.2 | 3.0 | 23.8 |
| *18* | 38 | 54 | F | 13.0 | 11.3 | 9.0 | 14.2 | 6.6 | 8.1 |
| *19* | 13 | 69 | F | 13.6 | 9.2 | 1.6 | 15.2 | 12.0 | 20.3 |
| *20* | 35 | 36 | M | 13.6 | 9.0 | 0.1 | 8.1 | 5.4 | 0.7 |
| *21* | 37 | 37.5 | F | 14.2 | 10.9 | 17.2 | 11.3 | 9.1 | 2.6 |
| *22* | 25 | 58.5 | M | 14.3 | 12.1 | n/a | 11.4 | 9.0 | 23.0 |
| *23* | 46 | 67.5 | M | 14.8 | 11.8 | 13.0 | 7.2 | 4.2 | 14.3 |
| *24* | 36 | 69 | F | 14.9 | 10.4 | 10.9 | 11.6 | 9.5 | 21.0 |
| *25* | 38 | 31.5 | F | 14.9 | 12.5 | 11.6 | 3.3 | 1.9 | 4.2 |
| *26* | 16 | 46.5 | F | 15.1 | 11.6 | n/a | 11.2 | 9.0 | 4.7 |
| *27* | 35 | 60 | M | 17.0 | 13.3 | n/a | 11.3 | 7.9 | n/a |
| *28* | 27 | 73.5 | M | 17.4 | 14.2 | 9.0 | 11.1 | 11.1 | n/a |
| *29* | 46 | 45 | F | 17.8 | 13.8 | 14.5 | 15.4 | 10.8 | n/a |
| *30* | 45 | 39 | F | 20.7 | 17.4 | 18.0 | 7.0 | 5.1 | n/a |
| *31* | 44 | 57 | F | 20.8 | 17.6 | n/a | 13.1 | 13.7 | 13.8 |
| *32* | 15 | 64.5 | M | 21.3 | 18.0 | n/a | 8.0 | 14.0 | 1.6 |
| *33* | 17 | 42 | M | 21.4 | 19.3 | n/a | 8.2 | 5.5 | 3.7 |
| *34* | 47 | 46.5 | M | 24.5 | 17.1 | 12.5 | 9.1 | 6.0 | 2.7 |
| *35* | 43 | 39 | F | 38.5 | 26.0 | n/a | 9.0 | 20.3 | 8.0 |

WBC- white blood cells; CRP- C-reactive protein; T0- day 1; T1- day 3; n/a- not applicable
